# Combined Coronary CT-Angiography and TAVI Planning: Utility of CT-FFR in Patients with Morphologically Ruled-Out Obstructive Coronary Artery Disease

**DOI:** 10.3390/jcm11051331

**Published:** 2022-02-28

**Authors:** Robin Fabian Gohmann, Patrick Seitz, Konrad Pawelka, Nicolas Majunke, Adrian Schug, Linda Heiser, Katharina Renatus, Steffen Desch, Philipp Lauten, David Holzhey, Thilo Noack, Johannes Wilde, Philipp Kiefer, Christian Krieghoff, Christian Lücke, Sebastian Ebel, Sebastian Gottschling, Michael A. Borger, Holger Thiele, Christoph Panknin, Mohamed Abdel-Wahab, Matthias Horn, Matthias Gutberlet

**Affiliations:** 1 Department of Diagnostic and Interventional Radiology, Heart Center Leipzig at University of Leipzig, Strümpellstr. 39, 04289 Leipzig, Germany; patrick.seitz@helios-gesundheit.de (P.S.); konrad.pawelka@helios-gesundheit.de (K.P.); adrian.schug@helios-gesundheit.de (A.S.); linda.heiser@helios-gesundheit.de (L.H.); katharina.renatus@helios-gesundheit.de (K.R.); christian.krieghoff@helios-gesundheit.de (C.K.); christian.luecke@helios-gesundheit.de (C.L.); sebastian.ebel@helios-gesundheit.de (S.E.); sebastian.gottschling@helios-gesundheit.de (S.G.); matthias.gutberlet@helios-gesundheit.de (M.G.); 2 Medical Faculty, University of Leipzig, Liebigstr. 27, 04103 Leipzig, Germany; 3 Department of Cardiology, Heart Center Leipzig at University of Leipzig, Strümpellstr. 39, 04289 Leipzig, Germany; nicolas.majunke@medizin.uni-leipzig.de (N.M.); steffen.desch@medizin.uni-leipzig.de (S.D.); philipp.lauten@me.com (P.L.); johannes.wilde@medizin.uni-leipzig.de (J.W.); holger.thiele@medizin.uni-leipzig.de (H.T.); mohamed.abdel-wahab@medizin.uni-leipzig.de (M.A.-W.); 4 Department of Cardiac Surgery, Heart Center Leipzig at University of Leipzig, Strümpellstr. 39, 04289 Leipzig, Germany; david.holzhey@helios-gesundheit.de (D.H.); thilo.noack@medizin.uni-leipzig.de (T.N.); philipp.kiefer@medizin.uni-leipzig.de (P.K.); michael.borger@medizin.uni-leipzig.de (M.A.B.); 5 Leipzig Heart Institute, Russenstr. 69a, 04289 Leipzig, Germany; 6 Siemens Healthcare GmbH, Henkestr. 127, 91052 Erlangen, Germany; christoph.panknin@siemens-healthineers.com; 7 Institute for Medical Informatics, Statistics and Epidemiology (IMISE), University of Leipzig, Härtelstr. 16-18, 04107 Leipzig, Germany; matthias.horn@imise.uni-leipzig.de

**Keywords:** aortic stenosis, computed tomography coronary angiography, coronary angiography, coronary artery disease, transcatheter aortic valve implantation, diagnostic accuracy, machine learning

## Abstract

***Background***: Coronary artery disease (CAD) is a frequent comorbidity in patients undergoing transcatheter aortic valve implantation (TAVI). If significant CAD can be excluded on coronary CT-angiography (cCTA), invasive coronary angiography (ICA) may be avoided. However, a high plaque burden may make the exclusion of CAD challenging, particularly for less experienced readers. The objective was to analyze the ability of machine learning (ML)-based CT-derived fractional flow reserve (CT-FFR) to correctly categorize cCTA studies without obstructive CAD acquired during pre-TAVI evaluation and to correlate recategorization to image quality and coronary artery calcium score (CAC). ***Methods***: In total, 116 patients without significant stenosis (≥50% diameter) on cCTA as part of pre-TAVI CT were included. Patients were examined with an electrocardiogram-gated CT scan of the heart and high-pitch scan of the torso. Patients were re-evaluated with ML-based CT-FFR (threshold = 0.80). The standard of reference was ICA. Image quality was assessed quantitatively and qualitatively. ***Results***: ML-based CT-FFR was successfully performed in 94.0% (109/116) of patients, including 436 vessels. With CT-FFR, 76/109 patients and 126/436 vessels were falsely categorized as having significant CAD. With CT-FFR 2/2 patients but no vessels initially falsely classified by cCTA were correctly recategorized as having significant CAD. Reclassification occurred predominantly in distal segments. Virtually no correlation was found between image quality or CAC. ***Conclusions***: Unselectively applied, CT-FFR may vastly increase the number of false positive ratings of CAD compared to morphological scoring. Recategorization was virtually independently from image quality or CAC and occurred predominantly in distal segments. It is unclear whether or not the reduced CT-FFR represent true pressure ratios and potentially signifies pathophysiology in patients with severe aortic stenosis.

## 1. Introduction

Coronary artery disease (CAD) is a frequent comorbidity in patients with severe aortic stenosis and is recommended to be excluded prior to transcatheter aortic valve implantation (TAVI) [1,2]. The exclusion of CAD has traditionally been undertaken via invasive coronary angiography (ICA); however, coronary computed angiography (cCTA) is now recommended to be considered as an alternative [1,3,4,5]. Numerous studies have confirmed the beneficial diagnostic profile of cCTA for the exclusion of CAD also in the cohort of patients considered for TAVI [6,7,8,9,10,11,12,13,14,15]. Noteworthy, when integrated as part of TAVI planning, cCTA may be performed without additional contrast medium and thus practically without additional risk to the patient [8]. However, the number of reported cCTAs before TAVI compared to the number of procedures performed seems disproportionately small [16]. This is striking as potentially any CT prior to TAVI performed in accordance with the guidelines [3] would be able to depict the coronary arteries technically robustly, and effectively constitute a cCTA.

A possible reason for the apparent reluctance to consistently report the coronary status prior to TAVI on CT may be the high plaque burden. Particularly, an elevated coronary artery calcium score (CAC) is likely to be a major contributing factor, being responsible for the high false positive rate and relatively low specificity of cCTA in this cohort [17]. CT-derived fractional flow reserve (CT-FFR) may increase the specificity and diagnostic accuracy, and has been described to do so also in patients prior to TAVI [18,19].

Furthermore, CT-FFR has been proposed to mitigate the apparent challenges in reading cCTA, when serving as a guide to morphological coronary analysis, improving interpretation speed and reader confidence, particularly for less experienced readers [20]. However, commercially available off-site solutions to CT-FFR are impractical for this purpose, as they require several hours for processing and are also costly [21,22,23]. Newer approaches to CT-FFR, namely those based on machine learning (ML), are much less computationally demanding and can be calculated on site in just a few seconds [24]. The results rendered by such newer algorithms are comparable to the more conventional approach of computational fluid dynamics [25]. Thus, ML-based CT-FFR could potentially be used as a guide to cCTA, or could even serve as a replacement of morphological cCTA analysis without the restrictions of time and costs related to off-site solutions.

In this study, we analyzed the ability of on-site ML-based CT-FFR to correctly categorize cCTA studies without morphological signs of obstructive CAD acquired during pre-TAVI evaluation. The secondary objective was to correlate recategorization to image quality measures and CAC.

## 2. Materials and Methods

### 2.1. Study Design

**Figure 1:**
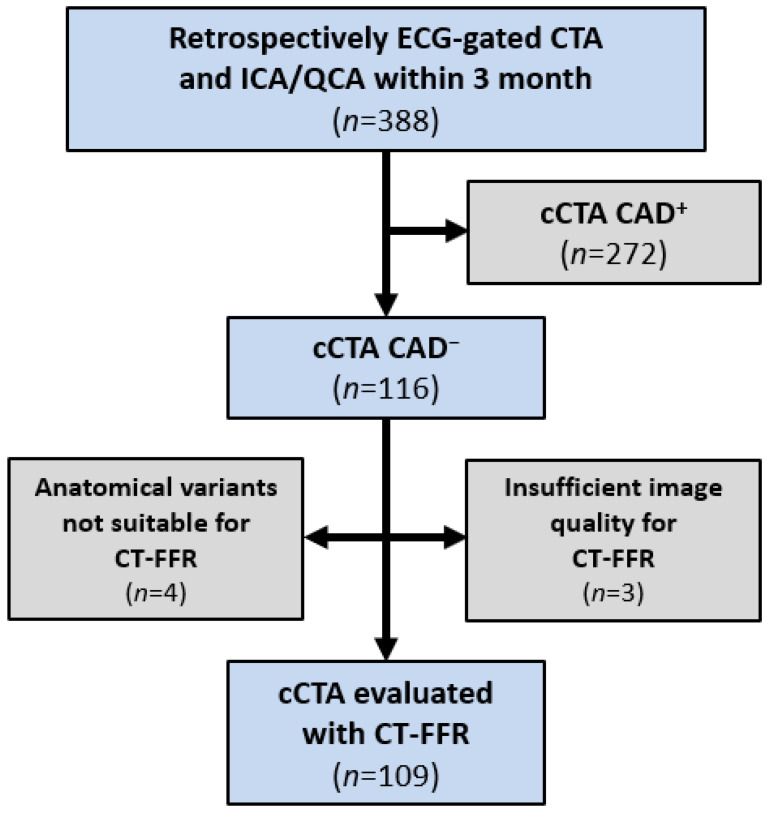
Flowchart of the study population according to diagnostics received. CAD^−^—no obstructive CAD on cCTA; CAD^+^—obstructive CAD (stenosis ≥50%) on cCTA; cCTA—coronary CT-angiography; CT-FFR—CT-derived fractional flow reserve; cCTA—coronary CT-angiography; ICA—invasive coronary angiography; QCA—quantitative coronary analysis.

The study design has previously been described in detail in [8]. Over a period of 7 months, 517 consecutive patients referred for CT prior to TAVI were screened. Overall, 388 patients had received the identical CT protocol and an ICA suitable for quantitative coronary analysis (QCA) within 3 months. Of these, 116 patients (116/388) had no morphological signs of obstructive CAD on cCTA (no stenosis of ≥50% diameter) and were included (Figure 1).

The study was conducted in compliance with the Declaration of Helsinki (Medical Association 2013). The local ethics committee approved the study and written informed consent was waived (reference number: 435/18-ek).

### 2.2. CT Acquisition

The scan protocol has previously been described in a more detailed manner in [8]. All patients were examined with the same scanner (Somatom Definition Flash; Siemens, Erlangen, Germany) and scan protocol. The scan protocol consisted of a nonenhanced prospectively ECG-triggered scan of the heart, and a retrospectively ECG-gated helical scan of the heart, immediately followed by high-pitch scan of the torso utilizing a single bolus of 70 mL contrast medium. No nitrates or beta blockers or other forms of patient-specific medication or adjustment were applied.

### 2.3. cCTA, ICA and QCA

cCTA had previously been evaluated morphologically for the presence of obstructive CAD (stenosis ≥50% diameter), separately for each segment according to the 18-segment model [8,26]. Results per vessel and patient were formed by considering the worst comprising segment, respectively. The standard of reference was ICA with QCA using the same threshold of ≥50% diameter.

### 2.4. Image Quality of cCTA and CAC

Image quality was assessed quantitatively and qualitatively as previously described [8,18]. Quantitatively, image quality was described as contrast opacification in the aortic sinus in Hounsfield units (HU) and as contrast to noise ratio (CNR) $=\frac{HU\;at\;aortic\;sinus-HU\;at\;interventricular\;septum}{noise\;of\;subcutaneous\;adipose\;tissue}$.

Qualitatively, contrast opacification, noise and artefacts were assessed and image quality was scored into one of the following four categories by considering the worst comprising component:

- 0 = nondiagnostic (excluded from this analysis, as CAD could not be excluded)
- 1 = diagnostic
- 2 = good
- 3 = excellent

CAC was determined using standard technique, separately for each of the four main coronary vessels [27]. Patients’ CACs was formed by summation.

### 2.5. CT-FFR

cCTA examinations without morphological signs of obstructive CAD (no stenosis ≥50% diameter) were re-evaluated with ML-based CT-FFR (cFFR version 3.2.0; Siemens, Erlangen, Germany; not commercially available) [24]. For this, epicardial coronary arteries with a minimum diameter of 1.5 mm were segmented and ML-based CT-FFR was computed. The time required for segmentation in this patient cohort was approximately 10 min on average and ranged from 5 to 30 min, depending on CNR and plaque burden. The actual computation of ML-based CT-FFR values was rapid (<5 s). CT-FFR measurements were taken for each segment of the 18-segment model at the junction of the middle and distal third within the respective segment [26]. Vessel and patient readings were formed by considering the respective minimum value. CT-FFR values ≤0.80 were considered to be indicative of hemodynamically significant CAD [28].

### 2.6. Statistical Analysis

Categorical variables are given as count and percentage; ordinal data is given as median and interquartile range (IQR). Continuous variables are expressed as mean and standard deviation (SD) when symmetrically distributed or as median and IQR for skewed distributions. Group comparisons were performed using independent two-sample *t*-tests for continuous symmetrically distributed variables and Mann–Whitney U tests for continuous skewed or ordinal data. For correlation analyses between recategorization status and potential disturbing variables, e.g., CAC or reduced quantitative and qualitative image quality, correlation coefficients and corresponding confidence intervals (CIs) were calculated. For this, rank-biserial correlation (between binary and continuous skewed or ordinal data) or point-biserial correlation (between binary and continuous symmetrically distributed variables) were applied. Correlation coefficients are denoted as $r_{rb}$ and $r_{pb}$, respectively. *p*-values correspond to the null hypothesis of the respective coefficient being zero. All tests were performed at a significance level of 5%. CIs are reported at a confidence level of 0.95.

Data curation and computation of inferential statistics were performed with spreadsheets (Microsoft Excel version 2010, Microsoft Corporation, Redmond, WA, USA). For further statistical analyses, R (v4.1.2, R Foundation for Statistical Computing, Vienna, Austria) was used.

## 3. Results

### 3.1. ML-Based CT-FFR

ML-based CT-FFR was successfully performed in 109 of the 116 (94.0%) cCTA exams without morphological signs of obstructive CAD. In 7 patients, ML-based CT-FFR was not feasible. Reasons for this were image quality hindering the seamless segmentation of the coronary tree (stitching artefacts or no single reconstruction with all segments depicted diagnostically at the same time; *n* = 3) or coronary anatomy, namely coronary anomalies or atypically dominant branches, outside of the boundaries the algorithm had been trained for [24], rendering errors during computation of CT-FFR (*n* = 4) (Figure 1).

When applied to all studies, ML-based CT-FFR recategorized 76 patients, 126 vessels and 186 segments from true negative (TN) to false positive (FP), respectively. Two patients initially categorized as false negative (FN) by cCTA were recategorized as true positive (TP) by CT-FFR. As the two vessels initially categorized as FN by cCTA were not recategorized by CT-FFR on vessel level, recategorization from FN to TP on patient level accrued because of a FP rating elsewhere in the coronary tree. As a consequence of the relatively high rate of recategorization from TN to FP, specificity and accuracy decreased by 71.0 and 67.9 percentage points on patient level, respectively. Further detail regarding the impact of CT-FFR on the diagnostic performance, including accuracy on patient, vessel and segment level, is shown in Table 1.

The rate of recategorization from TN with cCTA to FN with CT-FFR was low proximally in the vessels, with no or few recategorizations accruing in segments 1, 5, 6 and 11, and high in more distal segments. The number and rate of recategorizations for each segment and vessel as well as per patient are shown in Table 2.

### 3.2. Analysis According to Image Quality and CAC

CAC in the left anterior descending coronary artery (LAD) was significantly higher in patients recategorized as FP (CAC_LAD_: 42.6 (183.8); 118.0 (315.1); p=0.04). No further significant group differences in image quality parameters or CAC were noted between patients or vessels categorized as TN or FP (p≥0.10) (Table 3).

**Figure 2:**
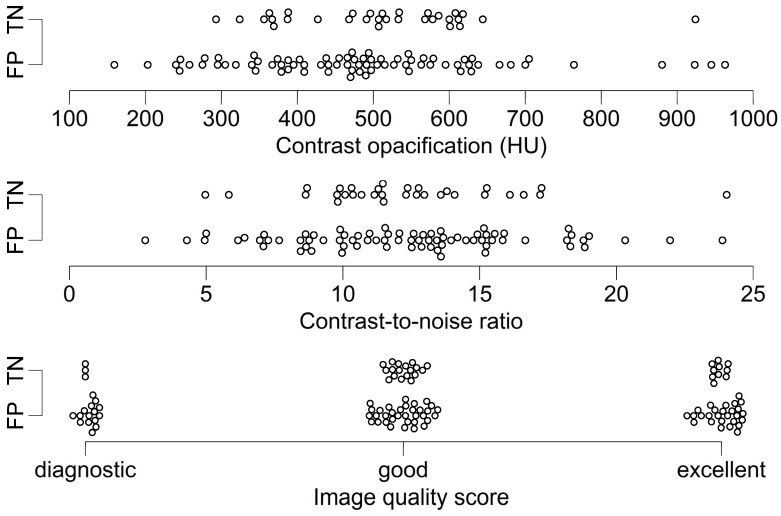
Dot-plot of cCTA image quality parameters and categorization according to ML-based CT-FFR of patients without morphological signs of obstructive CAD on cCTA. Recategorization of patients as false positive was independent of image quality, regardless of the concrete measure and accrued with comparable frequency in exams with diagnostic, good and exceptional image quality. The standard of reference was ICA with QCA. Thresholds were ≥50% diameter for cCTA and QCA and ≤0.80 for CT-FFR. Note—the two patients and vessels falsely categorized as negative with cCTA were excluded from this plot. cCTA—coronary CT-angiography; FN—false negative; FP—false positive; HU—Hounsfield units; ICA—invasive coronary angiography; ML—machine learning; QCA—quantitative coronary analysis.

Correlation between quantitative image quality parameters and recategorization from TN to FP was not significant (contrast opacification: $r_{pb}=0.07$, p=0.48; CNR: $r_{pb}=-0.007$, p=0.95). No dependence of recategorization and image quality score was found (image quality score 1: FP: 15, TN: 3; score 2: FP: 34, TN: 18; score 3: FP: 27, TN: 10; $r_{rb}=0.03$; p=0.73). For further detail see Table 3 and Figure 2.

**Figure 3:**
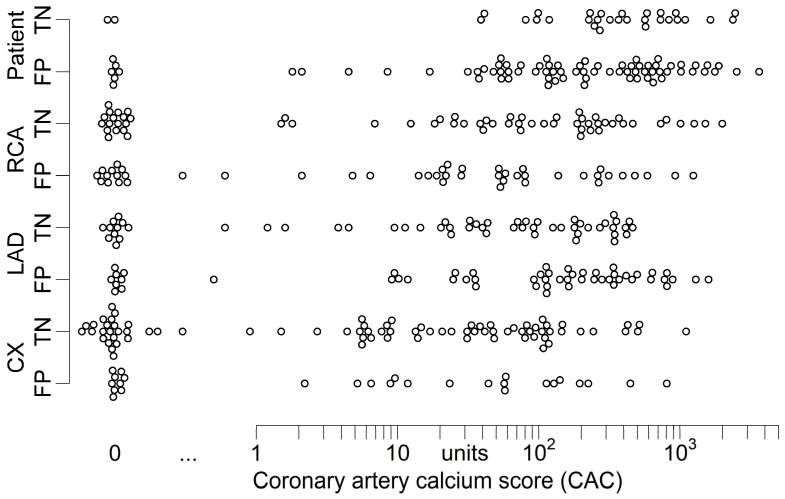
Dot-plot of patients’ and vessels’ CAC and categorization according to ML-based CT-FFR of cCTA studies without morphological signs of obstructive CAD on cCTA. Recategorization of patients and vessels was independent of CAC, occurring with roughly equal frequency with various extents of CAC. The standard of reference was ICA with QCA. Thresholds were ≥50% diameter for cCTA and QCA and ≤0.80 for CT-FFR. Note—the two patients and vessels falsely categorized as negative with cCTA were excluded from this plot. CAC was not available for all patients. CAC—coronary artery calcium score; cCTA—coronary CT-angiography; CX—circumflex artery, FN—false negative; FP—false positive; ICA—invasive coronary angiography; LAD—left anterior descending artery; ML—machine learning; RCA—right coronary artery; QCA—quantitative coronary analysis.

A weak negative correlation of CAC and recategorization to FP was found in the LAD ($r_{rb}=-0.20$; p=0.03). No further significant correlation of CAC and recategorization could be observed on patient or vessel level (patient: $r_{rb}=0.16$; right coronary artery: $r_{rb}=0.08$; circumflex artery: $r_{rb}=-0.01$; p≥0.10). Further details are shown in Table 3 and Figure 3.

## 4. Discussion

cCTA is a highly useful test for the detection and exclusion of obstructive CAD, characterized by high sensitivity and high negative predictive value. However, its specificity and positive predictive value are somewhat limited, and its diagnostic accuracy decreases with increasing plaque burden as frequently encountered in elderly patients [29], including patients prior to TAVI. Though CT-FFR has been shown to increase specificity and diagnostic accuracy also in this patient group when applied for specific lesions [18,19], interpreting cCTA and deciding between obstructive and non-obstructive CAD in patients with higher plaque burden may be challenging and requires experience. Therefore, it would be most convenient if CT-FFR could not only guide the semiquantitative interpretation of cCTA [20] but rather render a discrete value indicative of hemodynamically relevant CAD, thus making the more subjective morphological interpretation unnecessary. With this approach, CT-FFR could serve as a screening test and potentially facilitate decision making, particularly for less experienced readers or in more challenging cCTA examinations, e.g., in the group of patients prior to TAVI. To best illustrate the effect of an approach omitting the morphological evaluation, only exams previously acquitted of obstructive CAD were included in this analysis.

ML-based CT-FFR now enables such a workflow without the time or cost restraints applicable to earlier approaches [21,22,23] with the computation of CT-FFR on-site. However, our results demonstrate a false positive rate of 70% for CT-FFR in cCTA studies without morphological signs of obstructive CAD (no stenosis ≥50%). As a consequence, diagnostic accuracy was substantially degraded. Furthermore, the two patients formerly categorized as false negative with cCTA were only coincidentally recategorized as true positive on patient level because of false positive CT-FFR values elsewhere in the coronary tree (Table 1). Therefore, our results clearly discourage the unselective use of CT-FFR.

**Figure 4:**
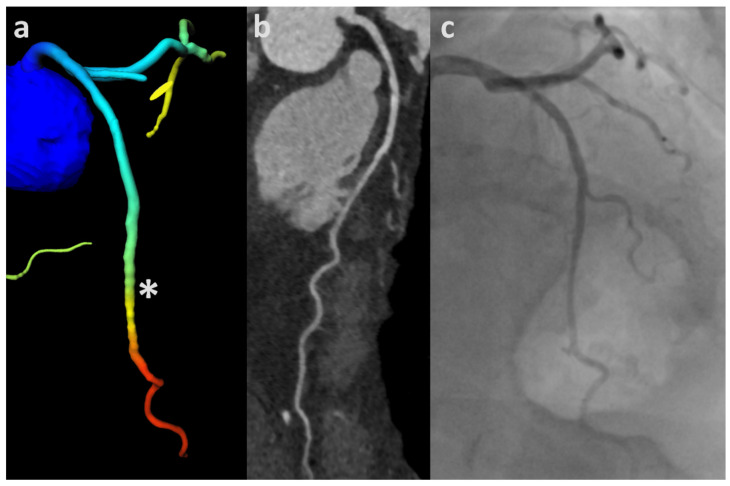
CT-FFR rendering values indicating hemodynamic significance with no apparent luminal narrowing on cCTA nor ICA: Mildly calcified left coronary artery (total CAC = 72 AU) with trifurcation into left anterior descending (LAD), left circumflex (LCX) and intermediate artery. CT-FFR values drop below 0.80 between the middle and distal segment (segment 6/7) (asterisk) (**a**). There is no discernable luminal obstruction on cCTA depicted as curved multiplanar reformation (**b**) nor on the corresponding projection of ICA (**c**). CAC—coronary artery calcium score; cCTA—coronary CT-angiography; CT-FFR—CT-derived fractional flow reserve; ICA—invasive coronary angiography.

The vast majority of false positive CT-FFR readings in regard to hemodynamic significant CAD were observed in more distal segments (Table 2, Figure 4). While it is generally recommended to consider CT-FFR values 1–2 cm distal to the lesion [28], this recommendation cannot be followed in patients with diffuse CAD or no discernable lesion whatsoever. Two previous studies have compared minimal CT-FFR measurements and measurements taken 2 cm distal to the lesion of interest. CT-FFR readings taken 2 cm distal to the lesion reduced the false positive rate by 44% and 54% with the same threshold, respectively [30,31]. While increased coronary artery resistance may be a product of diffuse atherosclerosis in the absence of obstructive CAD [32], we do not believe this to be the explanation for the majority of abnormal CT-FFR readings in our study cohort. Perhaps an imbalance between epicardial arterial volume and myocardial mass in patients with severe aortic stenosis caused by left ventricular hypertrophy may be a better explanation for the frequently low CT-FFR values in the distal coronary artery segments in our patient cohort [33,34].

The lack of patient preparation with nitroglycerine and beta blockers may degrade image quality and diagnostic accuracy of cCTA and CT-FFR [35,36]. However, we found no association between recategorization to false positive ratings and quantitatively or qualitatively assessed image quality in our study (Table 3 and Figure 2).

**Figure 5:**
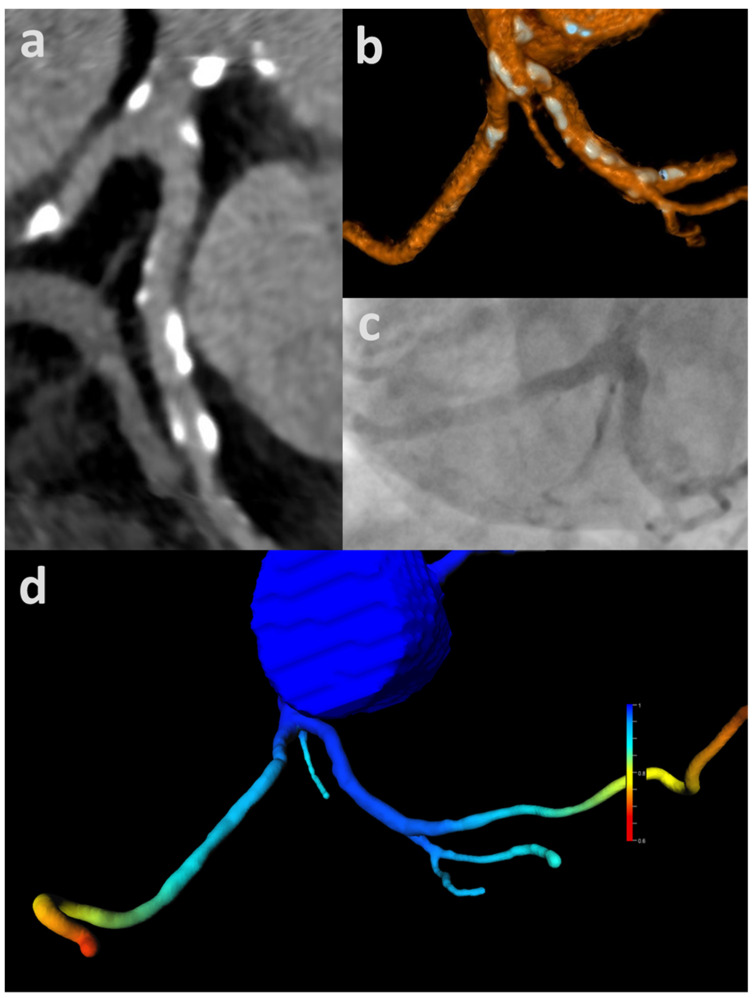
CT-FFR confirming negative cCTA: Heavily calcified left coronary artery (total CAC = 1834 AU) with trifurcation into left anterior descending (LAD), left circumflex (LCX) and intermediate artery without luminal obstruction depicted on cCTA as curved multiplanar reformation (**a**) and volume-rendered technique (**b**). The corresponding projection of invasive coronary angiography shows no stenosis (**c**). CT-FFR shows normal values well above 0.80 up to the distal vessels with a physiological drop-off of values only in the most distal runoffs (**d**). CAC—coronary artery calcium score; cCTA—coronary CT-angiography; CT-FFR—CT-derived fractional flow reserve. Adapted with permission from Gohmann et al. [8].

Similarly, CAC is well known to degrade diagnostic performance of both cCTA and CT-FFR [17,37,38]. Nevertheless, we only found a weak correlation between CAC and recategorization to FP for the LAD, prompted by very high CAC (CAC >400) (Table 3 and Figure 3). No correlation of CAC and recategorization was found for the remaining vessels or analysis on patient level (Table 3, Figure 3 and Figure 5).

The lack of relevant correlation between image quality measures and CAC may initially seem surprising. However, as this is an analysis of the performance of CT-FFR carried out on cCTA studies without morphological signs of obstructive CAD only, cCTA exams with impaired luminal delineation had already been excluded beforehand. Therefore, it is likely that the portrayal of the coronary artery lumen itself, rather than image quality or CAC, influence the values given by CT-FFR. It is thus likely that other factors are responsible for the false recategorization of studies without discrete stenosis in this patient cohort. As previously discussed, these factors may be diffuse atherosclerosis [32], or may potentially signify pathophysiology in patients with severe aortic stenosis, such as as an imbalance between epicardial arterial volume and myocardial mass [33,34].

All the same, if not applied lesion-specifically, CT-FFR may recategorize the majority of patients without morphological signs of obstructive CAD as false positive. Recategorization occurred much more frequently towards the distal portion of the coronary tree and was virtually independent of image quality measures or CAC. Thus, CT-FFR is not a suitable screening tool for CAD in the patient cohort prior to TAVI in this setting.

Physiologically, pressure gradients continuously decrease along any vessel, and, therefore, a discrete cut-off is perhaps not the best measure [39]. Potentially, other modes of measurement could prove to be better markers, for example, the relative change along the vessel per distance or between segments (delta) [40]. This would be interesting to explore also in this patient group.

### Limitations

This was a retrospective single-center study with an unusual selection of exams, namely cCTA studies of TAVI candidates without morphological signs of obstructive CAD. Thus, caution should be practiced when applying the results to different patient cohorts or types of cCTA exams.

It is well known that the lack of the administration of nitrates or beta blockers may degrade image quality and consequently diagnostic accuracy of both cCTA and CT-FFR [35,36]. However, as only exams without morphological signs of obstructive CAD were included, no exams with insufficient image quality for the delineation of the coronary lumina were included. This selection may explain the virtual lack of correlation of recategorization to image quality measures and CAC.

The standard of reference was ICA with QCA with a conservative threshold of ≥50% diameter stenosis. Despite being a very sensitive cut-off that will frequently not prompt therapy, it is morphological and it remains unclear whether or not the values obtained with CT-FFR are truly false low/positive at the location of measurement. Although very interesting, invasive functional measurements in patients with severe aortic stenosis and consequently altered hemodynamics remain controversial [41,42].

## 5. Conclusions

ML-based CT-FFR should carefully be used if utilized as a screening tool for CAD, e.g., for less experienced cCTA readers. Because, if unselectively applied, CT-FFR may vastly increase the number of false positive ratings of CAD compared to morphological scoring in patients before TAVI in the absence of obstructive lesions, particularly in distal segments. Recategorization to false positive was virtually independent of image quality or coronary artery calcium score. It is unclear whether or not the pathologically reduced CT-FFR represent true pressure ratios and potentially signifies pathophysiology in patients with severe aortic stenosis.

## Figures and Tables

**Table 1:** Comparison of cCTA and ML-based CT-FFR of patients without morphological signs of obstructive CAD.

	*n*	TP	TN	FP	FN	Sen.	Spe.	PPV	NPV	Acc.
Patients cCTA	109	0	107	0	2	0.0%	100.0%		98.2%	98.2%
Patients CT-FFR		2	31	76	0	100.0%	29.0%	2.6%	100.0%	30.3%
Difference Δ: patient level		2	−76	76	−2	+100.0%	−71.0%		+1.8%	−67.9%
Vessels cCTA	436	0	434	0	2	0.0%	100.0%		99.5%	99.5%
Vessels CT-FFR		0	308	126	2	0.0%	71.0%	0.0%	99.4%	70.6%
Difference Δ: vessel level		0	−126	126	0	0.0%	−29.0%		−0.2%	−28.9%
Segments cCTA	1456	0	1454	0	2	0.0%	100.0%		99.9%	99.9%
Segments CT-FFR		0	1268	186	2	0.0%	87.2%	0.0%	99.8%	87.1%
Difference Δ: segment level		0	−186	186	0	0.0%	−12.8%		0.0%	−12.8%

Results of coronary artery analysis with cCTA of a previous study [8] and analysis of ML-based CT-FFR against ICA/QCA on patient, vessel, and segment level. Thresholds for obstructive CAD were ≥50% diameter for cCTA and QCA and for hemodynamically significant CAD on CT-FFR ≤0.80, respectively. FN and TP results are ramifications from initial misclassification by cCTA. Acc.—accuracy; CAD^−^—negative for obstructive CAD; cCTA—coronary CT angiography; CT-FFR—CT-derived fractional flow reserve; FN—false negative; FP—false positive; ICA—invasive coronary angiography; ML—machine learning; NPV—negative predictive value; PPV—positive predictive value; Sen.—sensitivity; Spe.—specificity; TN—true negative; TP—true positive; QCA—quantitative coronary analysis.

**Table 2:** Recategorization of patients without morphological signs of obstructive CAD with ML-based CT-FFR according to location.

	*n*	FP (%)
**Pat.**	109	76 (70)
**RCA**	109	46 (42)
Seg. 1	109	0 (0)
Seg. 2	108	2 (2)
Seg. 3	101	13 (13)
Seg. 4	76	30 (39)
Seg. 16	80	26 (33)
**LM/Seg. 5**	109	0 (0)
**LAD**	109	53 (49)
Seg. 6	109	0 (0)
Seg. 7	109	9 (8)
Seg. 8	108	50 (46)
Seg. 9	88	11 (13)
Seg. 10	56	11 (20)
Seg. 17	34	3 (9)
**CX**	109	27 (25)
Seg. 11	109	1 (1)
Seg. 12	88	7 (8)
Seg. 13	90	6 (7)
Seg. 14	58	7 (12)
Seg. 15	11	4 (36)
Seg. 18	13	6 (46)

Recategorization with ML-based CT-FFR of patients without morphological signs of obstructive CAD on cCTA against ICA/QCA on patient, vessel and segment level. Note: 7 patients were excluded because of image quality or anatomic variants not suitable for ML-based CT-FFR. Thresholds for obstructive CAD were ≥50% diameter for cCTA and QCA and ≤0.80 for CT-FFR. Segment definition according to the 18-segment model [26]. CAD—coronary artery disease; cCTA—coronary CT angiography; CT-FFR—CT-derived fractional flow reserve; FP—false positive; ICA—invasive coronary angiography; Seg.—segment; QCA—quantitative coronary analysis.

**Table 3:** Group comparison and correlation between recategorization status and image quality parameters or CAC.

Variables	TN (*n* = 31)	FP (*n* = 76)	*p*	Correlation Coefficient	CI	*p*
Contrast opacification (HU)	510.9 ± 125.8	487.3 ± 165.2	0.43	0.07	−0.12, 0.26	0.48
CNR	12.33 ± 3.67	12.38 ± 4.19	0.94	−0.007	−0.20, 0.18	0.95
Image quality score	2 (1)	2 (1)	0.74	0.03	−0.15, 0.21	0.73
CAC_Patient_	343.4 (584.1)	189.6 (538.1)	0.10	0.16	−0.03, 0.34	0.10
CAC_RCA_	47.2 (225.5)	22.3 (80.1)	0.39	0.08	−0.11, 0.27	0.39
CAC_LAD_	42.6 (183.8)	118.0 (315.1)	0.04	−0.20	−0.38, −0.01	0.03
CAC_CX_	9.0 (80.4)	9.6 (85.9)	0.91	−0.01	−0.21, 0.19	0.91

Group comparison and correlation measures between image quality parameters or CAC on patient and vessel level and recategorization status from true negative (TN) to false positive (FP) with ML-based CT-FFR of patients without morphological signs of obstructive CAC. Thresholds for obstructive CAD were ≥50% diameter for cCTA and QCA and ≤0.80 for CT-FFR. For group comparisons (TN vs. FP) median (and IQR) (image quality score and CAC) or means ± SD (contrast opacification and CNR) are given for both groups, and Mann–Whitney U tests and t-tests were performed, respectively. Correlation coefficients and corresponding CIs were calculated using rank-biserial correlation (between recategorization status and image quality score or CAC) or point-biserial correlation (between recategorization status and contrast opacification or CNR). *p*-values of correlation coefficients correspond to the null hypothesis of the respective coefficient being zero. CAC—coronary artery calcium scoring; CAD—coronary artery disease; cCTA—coronary CT angiography; CI—confidence interval; CNR—contrast to noise ratio; CT-FFR—CT-derived fractional flow reserve; CX—circumflex artery; FP—false positive; HU—Hounsfield units; IQR—interquartile range; LAD—left anterior descending artery; RCA—right coronary artery; TN—true negative; SD—standard deviation; QCA—quantitative coronary analysis.

## Data Availability

The datasets generated and/or analyzed during the current study are not publicly available due to German Data Protection laws but are available from the corresponding author on reasonable request after approval of the local ethics committee and data safety board.
